# Human multipotent adult progenitor cell-conditioned medium improves wound healing through modulating inflammation and angiogenesis in mice

**DOI:** 10.1186/s13287-020-01819-z

**Published:** 2020-07-17

**Authors:** Parinaz Ahangar, Stuart J. Mills, Louise E. Smith, Xanthe L. Strudwick, Anthony E. Ting, Bart Vaes, Allison J. Cowin

**Affiliations:** 1 Future Industries Institute, University of South Australia, Adelaide, SA 5000 Australia; 2 Cell Therapy Manufacturing Cooperative Research Centre, Adelaide, SA 5000 Australia; 3 Athersys, Inc., Cleveland, OH USA; 4 ReGenesys BVBA, Bio-Incubator Leuven, Gaston Geenslaan 1, 3001 Heverlee, Belgium

**Keywords:** Wound healing, Multipotent adult progenitor cells, Secretome, Conditioned medium, Inflammation, Angiogenesis

## Abstract

**Background:**

Stem cell therapies have been widely investigated for their healing effects. However, the translation of these therapies has been hampered by the requirement to deliver live allogeneic or autologous cells directly to the wound in a clinical setting. Multipotent adult progenitor cells (MAPC® cells) are a subpopulation of bone marrow-derived adherent stem cells that secrete a wide range of factors known to accelerate the wound healing process. The aim of this study was to determine the impact of MAPC cells secretome on healing outcomes without the presence of MAPC cells.

**Methods:**

The effect of MAPC-conditioned medium (MAPC-CM) on the capacity of keratinocytes, fibroblasts and endothelial cells to migrate and proliferate was determined in vitro using scratch wound closure and WST1 assay, respectively. The effect of MAPC-CM on collagen deposition and angiogenesis was also assessed using in vitro methods. Additionally, two excisional wounds were created on the dorsal surface of mice (*n* = 8/group) and 100 μL of 20× MAPC-CM were intradermally injected to the wound margins. Wound tissues were collected at 3, 7 and 14 days post-wounding and stained with H&E for microscopic analysis. Immunohistochemistry was performed to investigate inflammation, angiogenesis and collagen deposition in the wounds.

**Results:**

Skin fibroblasts, keratinocytes and endothelial cells treated with MAPC-CM all showed improved rates of scratch closure and increased cellular proliferation. Moreover, fibroblasts treated with MAPC-CM deposited more collagens I and III and endothelial cells treated with MAPC-CM showed increased capillary tube formation. Murine excisional wounds intradermally injected with MAPC-CM showed a significant reduction in the wound area and an increase in the rate of reepithelialisation. The results also showed that inflammatory cell infiltration was decreased while an increase in angiogenesis, as well as collagens I and III expressions, was observed.

**Conclusion:**

These findings suggest that factors produced by MAPC cells can have an important effect on cutaneous wound healing by affecting skin cell proliferation and migration, balancing inflammation and improving the formation of extracellular matrix and angiogenesis. Development of stem cell-free therapy for the treatment of wounds may be a more clinically translatable approach for improving healing outcomes.

## Introduction

Wound healing is a well-coordinated process in which various cell types receive external signals causing them to proliferate, migrate, differentiate and synthesise proteins to restore the multilayered tissue of skin [1]. During wound healing, fibroblasts from the surrounding dermal layer proliferate and migrate into the wound site. Fibroblasts in the wound area deposit extracellular matrix (ECM) into the wound bed, which results in the formation of new granulation tissue [2]. Simultaneously, endothelial cells migrate into the wound bed and create tube-like structures, which form the foundation of new blood vessels. Finally, the skin barrier is restored during the re-epithelialisation process where keratinocytes proliferate and migrate across the wound bed to form the neo-epidermis [3]. Any dysfunction in the cutaneous wound healing process such as prolonged inflammation, delayed proliferation and/or excessive collagen deposition results in the formation of chronic wounds and additional scarring in human adults [4].

Therapeutic potential of stem cells has been investigated for the repair and regeneration of damaged tissues and both preclinical and clinical trials have shown great promise for the use of stem cells in wound healing improvement [5, 6]. However, the development of stem cell therapies for the treatment of wounds has been hampered by the requirement to deliver large numbers of live, functional cells to patients [7].

Stem cell differentiation and direct incorporation into regenerating tissues were speculated to be the primary mechanisms of mesenchymal stem cell (MSC) actions [8]. However, several cases have demonstrated that frequency of stem cell engraftment and the number of newly generated cells, either by differentiation or by cell fusion, appears to be too low to explain significant effects achieved by stem cells [9]. Proteomic analysis of stem cell conditioned media indicates that stem cells secrete a wide range of biomolecules which can contribute to tissue regeneration including mRNAs, active lipids, growth factors and cytokines [10]. Therefore, the paracrine signalling of stem cells has been suggested as the main mechanism for the actions of stem cells [9]. Evidence from several in vitro and in vivo studies suggest that beneficial effects of stem cell therapies on wound healing are achieved via their paracrine effects on skin cells. This increases the rate of proliferation and migration and functionality in resident immune cells, keratinocytes, fibroblasts and endothelial cells [11].

Multipotent adult progenitor cells (MAPC cells) are a sub-set of adherent stem cells that have outstanding plasticity and self-renew ability [12]. These cells initially were derived from adult bone marrow [12] but have also been isolated from brain and muscle tissues [13]. In comparison with MSCs, MAPC cells have been considered as a more biologically primitive population with greater differentiation and proliferation potential [14]. Previously published studies have confirmed MAPC cells as a distinct cell-type separate from MSCs with different phenotype, function, differentiation and expansion capacity, required culture conditions and transcriptional profile [12, 15–17]. Furthermore, MAPC cells and MSCs possess distinct secretomes based on their surrounding environments [18]. Analysis of these secretomes has demonstrated that MAPC cells respond to various stimuli by secreting a wide range of growth factors and cytokines which affect processes such as angiogenesis, immune function, fibrosis, and apoptosis, all key processes during wound healing [19].

This paper aimed to investigate whether human MAPC-conditioned medium (MAPC-CM) could accelerate cutaneous wound healing in vitro and in vivo. First, proteins secreted by MAPC cells were identified using a bead array assay. Second, the effect of MAPC-CM on proliferation, migration and functionality of cutaneous cells including keratinocytes, fibroblasts and endothelial cells was determined using in vitro methods. Finally, the effect of MAPC-CM on excisional wound healing was investigated using a murine wound healing model. In order to evaluate this effect, macroscopic and microscopic assessments were employed. Moreover, the impact of this treatment on inflammation, angiogenesis and collagen deposition was determined in the healing wounds.

## Materials and methods

### Cell culture

Human MAPC cells were provided by ReGenesys BVBA (Heverlee, Belgium). MAPC cells were isolated from a single bone marrow aspirate, obtained with consent from a healthy donor and subsequently expanded on a Quantum cell expansion system (Terumo BCT, Lakewood, USA) according to previously described methods [16]. Cells were further expanded on fibronectin-coated plastic tissue culture flasks. Cell cultures were maintained under low oxygen tension in a humidified atmosphere of 5% CO_2_. Cells were cultured to sub-confluence in MAPC culture media (low-glucose DMEM (Thermo Fisher Scientific, VIC, Australia) supplemented with foetal bovine serum (FBS, Atlas Biologicals, Fort Collins, USA), ITS liquid media supplement (Sigma-Aldrich, NSW, Australia), MCDB (Sigma-Aldrich, NSW, Australia), platelet-derived growth factor (R&D Systems, Minneapolis, USA), epidermal growth factor (R&D Systems), dexamethasone (Sigma-Aldrich, NSW, Australia), penicillin/streptomycin (Invitrogen, CA, USA), 2-Phospho-L-ascorbic acid (Sigma-Aldrich, NSW, Australia) and linoleic acid–albumin (Invitrogen, CA, USA)). Cells were passaged every 3–4 days and harvested using trypsin/EDTA (Invitrogen, CA, USA). Transcriptome analysis confirmed that MAPC cells are distinct cell population from MSCs at the point of use (Supplementary Information and Fig. S1).

Human foreskin fibroblasts (HFFs, CellBank Australia, NSW, Australia) and human immortalised keratinocytes (HaCaTs, ATCC, Virginia, USA) were cultured in low-glucose DMEM medium (Thermo Fisher Scientific, VIC, Australia) with 2 mM L-glutamine, 10% FBS (Thermo Fisher Scientific, VIC, Australia) and 100 U/mL penicillin/streptomycin (Lonza, Basel, Switzerland). Human dermal microvascular endothelial cells (HDMECs, PromoCell, Heidelberg, Germany) were grown in endothelial cell growth medium MV2 (PromoCell, Heidelberg, Germany) with 2 mM L-glutamine, 10% FBS and 100 U/mL penicillin/streptomycin (Lonza, Basel, Switzerland). These three cell types were incubated at 5% CO_2_, 37 °C and 95% humidity.

### MAPC-conditioned medium collection

To harvest MAPC-CM, 2 × 10^3^ cells per cm^2^ were seeded into T75 flasks. Upon reaching 70% confluency, MAPC medium was removed, flasks were washed twice with phosphate-buffered saline (PBS) and 10 mL FBS-free DMEM medium was added to the flasks. Following 24 h incubation under hypoxia conditions, the conditioned medium was collected from each flask. To obtain consistent small batches of CM, the conditioned media of MAPC cells in 20 flasks were combined, centrifuged at 350 g for 10 min and sterilised using 0.22 μm filters. For in vivo applications, the conditioned medium was concentrated 20× using GE Vivaspin™ protein concentrator spin columns (Bio-strategy, VIC, Australia). Aliquots were kept in − 80 °C freezer until use. In all experiments, fresh FBS-supplemented DMEM with 10% FBS was used as the positive control while fresh FBS-free DMEM medium was used as the negative control.

### Bead array assay

A bead-based immunoassay was employed to detect and quantify the concentration of soluble proteins in MAPC-CM including growth factors, adhesion molecules and inflammatory cytokines. The experiment was performed according to the manufacturer’s protocols (BioLegend’s LEGENDplex™, CA, USA). Briefly, human MAPC cells were cultured for at least two passages under normal culture conditions. Prior to the collection of conditioned medium, MAPC cells (75–85% confluent) were cultured in FBS-free DMEM for 24 h in the absence of any exogenous stimuli. Then, the collected conditioned medium was concentrated 20× using GE Vivaspin™ protein concentrator spin columns. Concentrated conditioned medium and standards were incubated with antibody-conjugated capture beads for each protein for 2 h with shaking. Following binding of the capture beads with their target proteins, biotinylated detection antibodies were added to the wells and incubated for an hour with shaking. Streptavidin phycoerythrin (SA-PE) was added to the wells and incubated for 30 min. After washing with SA-PE, the wells were loaded with wash buffer and read using a BD LSRFortessa™ flow cytometer (BD Bioscience, CA, USA).

### Cell proliferation assay

The effect of MAPC-CM on the proliferation of HFFs, HaCaTs and HDMECs was analysed using the WST-1 (2-(4-iodophenyl)-3-(4-nitrophenyl)-5-(2,4-disulfophenyl)-2H-tetrazolium monosodium salt) proliferation assay according to the manufacturer’s protocols (Roche Applied Science, Bavaria, Germany). In brief, cells were plated onto 96-well plates at the density of 5 × 10^3^ cells/well in their own recommended culture conditions. When they reached 25–35% confluence, cells were washed with PBS to remove media and the media were replaced with either FBS-free DMEM (negative control), DMEM with 10% FBS (positive control) or MAPC-CM. Following 24 h incubation, WST-1 (10%) was added to each plate and the absorbance was measured at 460 nm using a microplate reader (Tecan Group, Mannedorf, Switzerland).

### Scratch wound closure assay

Migration of dermal fibroblasts, keratinocytes and endothelial cells in MAPC-CM was assessed using a scratch wound closure assay. To conduct this assay, cells were seeded onto 96-well plates at the density of 3 × 10^4^ cells/well in their own recommended media and maintained until reaching 80–90% confluency. Cell monolayers were scratched using a wound maker™ (Essen bioscience, MI, USA). After scratching, wells were washed twice using PBS and their culture media was replaced with either FBS-free DMEM (negative control), DMEM with 10% FBS (positive control) or MAPC-CM. Plates were then placed in the IncuCyte (Essen bioscience, MI, USA) to record the scratch wound closure by taking images immediately after scratching and every 3 h until complete wound closure. For the study on the MAPC-CM, the remaining scratch area of each well was calculated and presented as the extent of wound closure.

### Collagen I and III staining of fibroblasts

Collagens I and III were determined using a 96-well immunohistochemistry assay [20]. HFFs were seeded in DMEM with 10% FBS at 5 × 10^4^ cells/well into 96-well plates. The medium was replaced with test reagents after 24 h. Plates were left in the incubator for 60 h to allow collagens to be deposited. Cells were washed with PBS, fixed and permeabilised in ice-cold methanol (− 20 °C). Cells were then treated with Tween 20 (0.5%) in PBS for 10 min before incubating in 3% normal goat serum (NGS, Thermo Fisher Scientific, VIC, Australia) for 30 min and finally incubating with rabbit anti-human collagen type I antibody (5 μg/mL, Rockland Immunochemicals, USA) and rabbit anti-human collagen type III Antibody (5 μg/mL, Rockland Immunochemicals, USA) at room temperature for 2 h. Secondary antibody goat anti-rabbit IgG Alexa Fluor 488 (5 μg/mL, Invitrogen, CA, USA) was added to cells for an hour. Nuclear fluorescent dye (DAPI, (4′, 6-diamidino-2-phenylindole)) was added prior to imaging. The plate was then imaged using an Olympus IX83 fluorescence microscope and the fluorescence intensity of the wells was measured in order to determine the expression of collagens I and III.

### Matrigel tube formation assay

An in vitro tube formation assay was employed in order to examine the possible effect of MAPC-CM on blood vessel formation. This assay investigates the ability of human endothelial cells to form tube-like networks and tube sprouts after incubation in MAPC-CM. Ten microlitres growth factor reduced Matrigel (Corning Life Science, New York, USA) was added to each well of Ibidi u-slide angiogenesis 15-well plates (ibidi GmbH, Bavaria, Germany), which then was incubated at 37 °C for 30 min. HDMECs (1.5 × 10^4^) suspended in 50 μL of either FBS-free DMEM (negative control), DMEM with 10% FBS (positive control) or MAPC-CM were seeded in each well and images were taken every 3 h using an Olympus IX81 microscope (Olympus, Tokyo, Japan). The number of tubes in each well was counted every 3 h until all tubes were degraded.

### Murine model of excisional wound healing

Female BALB/c mice 10–12 weeks old were used for the study. Two 6 mm^2^ full-thickness dorsal excisional wounds were created 5 mm either side of the midline and 10 mm from the base of the skull. One hundred microlitres of 20× concentrated conditioned medium was injected at four sites into the margins of each wound. One hundred microlitres FBS-free DMEM was injected at four sites into the margins of wounds in the control group (*n* = 8/group). Digital photographs of the wounds were taken for macroscopic analysis of wound healing and wounds were harvested at days 3, 7 and 14 post-injury for histological and immunohistochemical analysis.

### Histological and immunohistochemistry analyses of wound tissues

Histological sections (4 μm) from paraffin-embedded fixed tissues were stained with haematoxylin and eosin (H&E) for microscopic analysis of wound healing. The sections were also stained with Masson’s trichrome stain for total collagen analysis. Moreover, sections were subjected to immunohistochemistry, similar to previous studies [21], following antigen retrieval. Tissue sections were blocked in 3% blocking NGS for 30 min at room temperature, incubated with rabbit anti-human collagen type I (5 μg/mL, Rockland Immunochemicals, Pennsylvania, USA), rabbit anti-human collagen type III (5 μg/mL, Rockland Immunochemicals, Pennsylvania, USA), rat neutrophil marker (NIMP-R14, 0.5 μg/mL, Santa Cruz Biotechnology, Texas, USA) and rabbit anti-CD31 (0.2 μg/mL, Abcam, Cambridge, UK), F4/80 (5 μg/mL, Bio-Rad, NSW, Australia), YM-1 (0.37 μg/mL, StemCell technologies, Vancouver, Canada) antibodies overnight at 4 °C. Subsequently, tissue sections were stained with secondary goat anti-rabbit IgG, Alexa Fluor 488, goat anti-rabbit IgG, Alexa Fluor 568 or goat anti-rat IgG Alexa Fluor 488 (5 μg/mL, Invitrogen, CA, USA). To visualise nuclei and overall tissue architecture, sections were stained for 5 min in 4′,6-diamidino-2-phenylindole (DAPI, 1:5000 of 1 mg/mL stock). Sections were imaged with Olympus IX81 microscope (Olympus, Tokyo, Japan).

### Statistical analysis

The results are presented as mean ± SEM. Data analysis was performed using GraphPad Prism (Graphpad, CA, USA). One-way and two-way analysis of variance (ANOVA) tests were used for multiple comparisons followed by Tukey and Bonferroni post-tests. Data are representative of the means of at least three independent experiments and values. A value of *p* < 0.05 was set for the significance value.

## Results

### Secretome analysis of MAPC cells and identification of proteins correlated to wound healing

In order to identify proteins secreted into the conditioned media by MAPC cells, bead array assays were employed. This assay was conducted to quantify a number of proteins associated with wound healing including growth factors, inflammatory cytokines and adhesion molecules. The concentration of secreted proteins found in conditioned media of MAPC cells is shown in Table 1 along with their role in wound healing. Note that no protein was detected in FBS-free DMEM.

**Table 1:** MAPC cell protein secretome. Functionally distinct classes of molecules identified by bead array assays

	ID/concentration (pg/mL)	Description/function
ECM proteins	Matrix metalloproteinase (MMP-1)/(29,838 ± 385)	Involved in collagen degradation, ECM remodelling cell migration [22] and angiogenesis [23].
	Tissue inhibitor of metalloproteinase (TIMP-1)/(> 771,464)	Inhibitor of MMPs and regulator of cell migration [22].
Growth factors and modulatory proteins	Hepatocyte growth factor (HGF)/(1817 ± 38)	Accelerates re-epithelialisation. Suppresses inflammation. Promote angiogenesis [24]
	Vascular endothelial growth factor (VEGF) (725 ± 42)	Proangiogenic mediator [25].
	Fibroblast growth factor-2 (FGF-2)/(172 ± 55)	Promotes proliferation and migration of fibroblasts and affect collagen deposition [26]. Contributes to the collagen maturation [27].
Adhesion Molecules	Vascular cell adhesion protein (VCAM-1)/(32,641 ± 1538)	Cell adhesion molecule. Involves in inflammation, cell proliferation and migration [28].
	CD166 (ALCAM)/(89,555 ± 955)	Regulator of T cells and MMP activity [29].
	CD44/(9344 ± 938)	Regulator of cell migration, survival and differentiation [30].
Inflammatory cytokines	Monocyte Chemoattractant Protein-1 (MCP-1)/(17 ± 1)	Controls the migration and recruitment of monocytes/macrophages during inflammation [31].
	Interleukins (IL): IL-8 (262 ± 110), IL-2 (21 ± 2), IL-1β (9 ± 0)	Pro-inflammatory cytokines [32].
	IL-6 (1150 ± 414)	A pro-inflammatory cytokine and an anti-inflammatory cytokine [33]

### MAPC-CM increases the proliferation and migration of fibroblasts, keratinocytes and endothelial cells

**Fig. 1:**
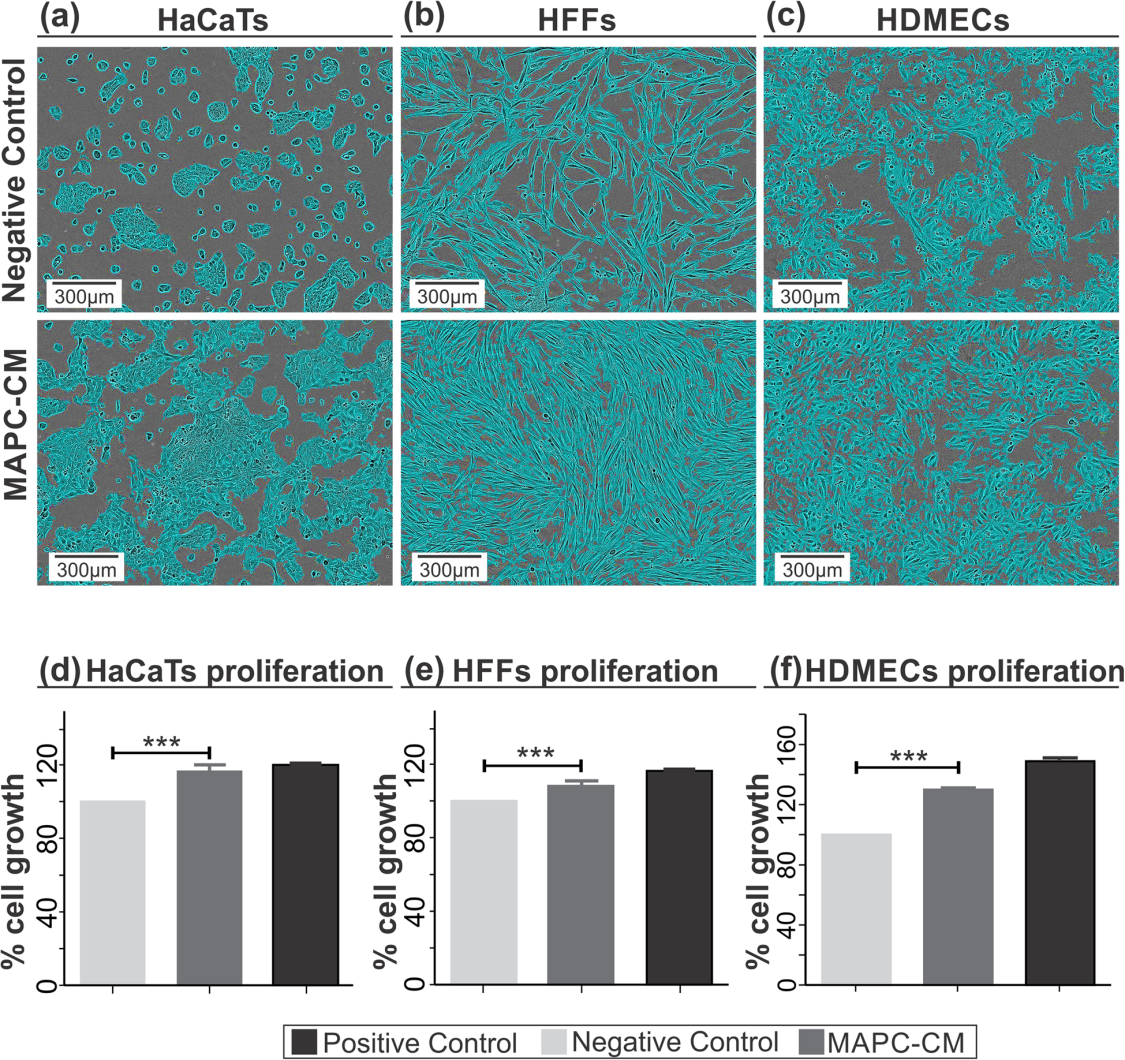
Effect of MAPC-CM on the proliferation of skin cells. Representative images of all three skin cell types 24 h after treatment with MAPC-CM versus negative control media. Cells are masked with confluency mask of Incucyte zoom software (**a**–**c**). The proliferation was quantified by WST-1 assay which demonstrates increased proliferation in MAPC-CM-treated group compared to the negative control group in **d** HaCaTs, **e** HFFs and **f** HDMECs. Values are mean ± SEM of 3 independent experiments. *n* = 6. Statistical significance calculated by one-way ANOVA followed by Tukey’s test, where * = *p* < 0.05, ** = *p* < 0.01, *** = *p* < 0.001, ns = non-significant

To measure the effect of MAPC-CM on skin cells proliferation, a WST-1 assay was used. As shown in Fig. 1, the proliferation of HaCaTs was significantly increased by 17.4% following 24 h of treatment with MAPC-CM in comparison to the FBS-free DMEM (negative control) (Fig. 1a, d). Proliferation of MAPC-CM-treated HFFs was also increased by 8.8% (Fig. 1b, e). HDMECs similarly showed elevated proliferative ability with an increase of 29.3% over the negative control (Fig. 1c, f).

**Fig. 2:**
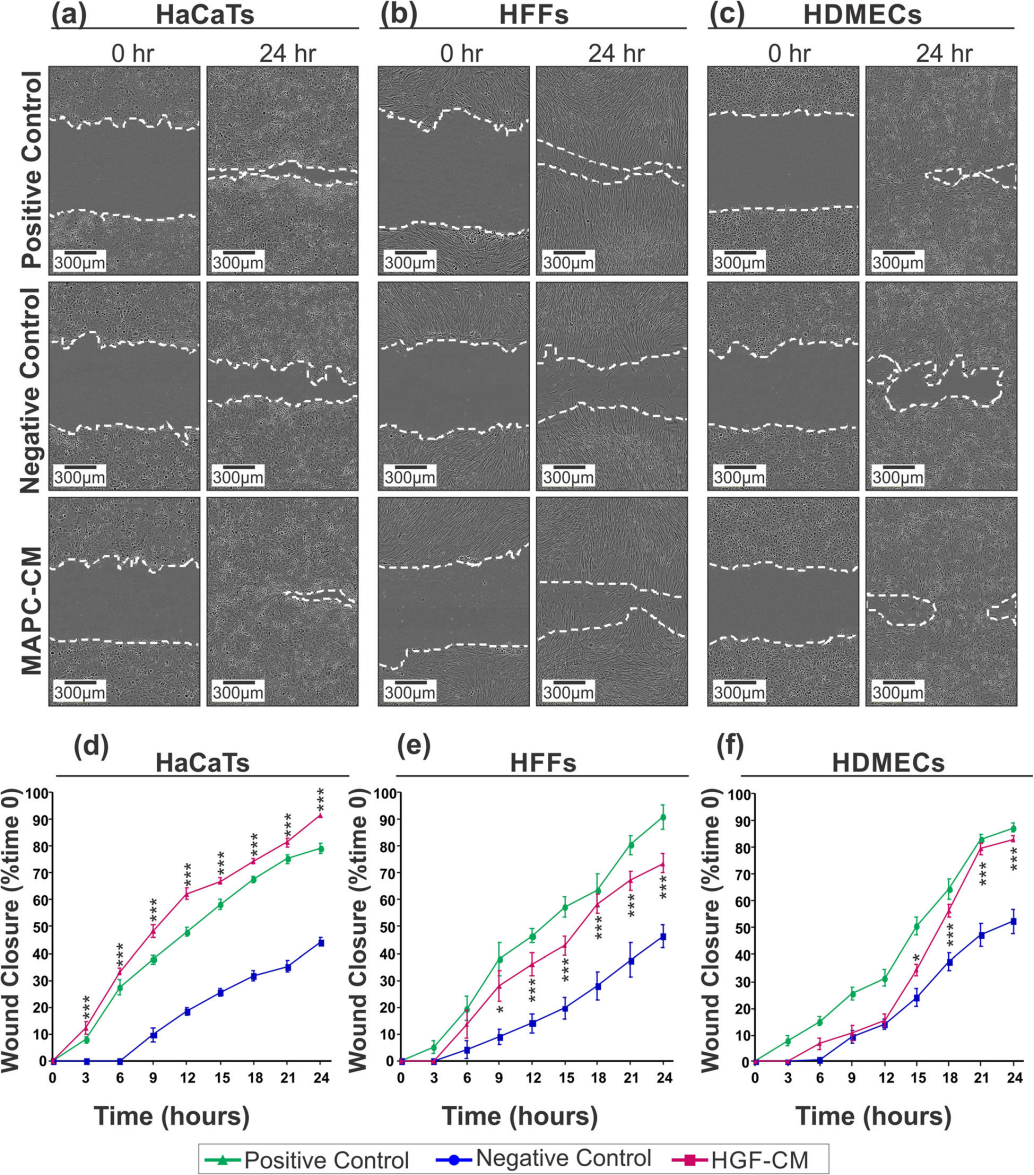
Effect of MAPC-CM on the migration of skin cells. Representative images of scratch assays show scratches immediately after the scratches had been made and then after 24 h in the presence of MAPC-CM versus positive and negative control media. The percentage signifies the extent of wound closure 24 h after making scratches, compared to the initial scratch area and measured using Incucyte zoom and ImageJ software (**a**–**c**). There was a significant increase in the extent of wound closure in MAPC-CM-treated group than in the negative control group in **d** HaCaTs, **e** HFFs and **f** HDMECs. Values are mean ± SEM of 3 independent experiments. *n* = 6. Statistical significance calculated by one-way ANOVA followed by Tukey’s test, where * = *p* < 0.05, ** = *p* < 0.01, *** = *p* < 0.001, ns = non-significant

In order to determine the effect of MAPC-CM on the migration ability of skin cells, a scratch wound closure assay was used. Migration of HaCaTs into the scratch area was significantly enhanced from the first time point (3 h) after scratch, while cells in FBS-free DMEM (negative control) had not started to migrate (Fig. 2a, d). Migration of HaCaTs into the scratched area was significantly increased at each time point compared to the negative control. This increase was even significantly higher than the DMEM + 10% FBS (positive control) at each time point (Fig. 2a, d). Migration ability of HFFs was also significantly enhanced following 9 h of treatment with MAPC-CM compared to negative control medium (Fig. 2b, e). Similarly, migration of HDMECs was enhanced in the presence of MAPC-CM, which was statistically significant 15 h after scratching (Fig. 2c, f).

### MAPC-CM encourages tube formation of endothelial cells and increases the halftime life of formed tubes

**Fig. 3:**
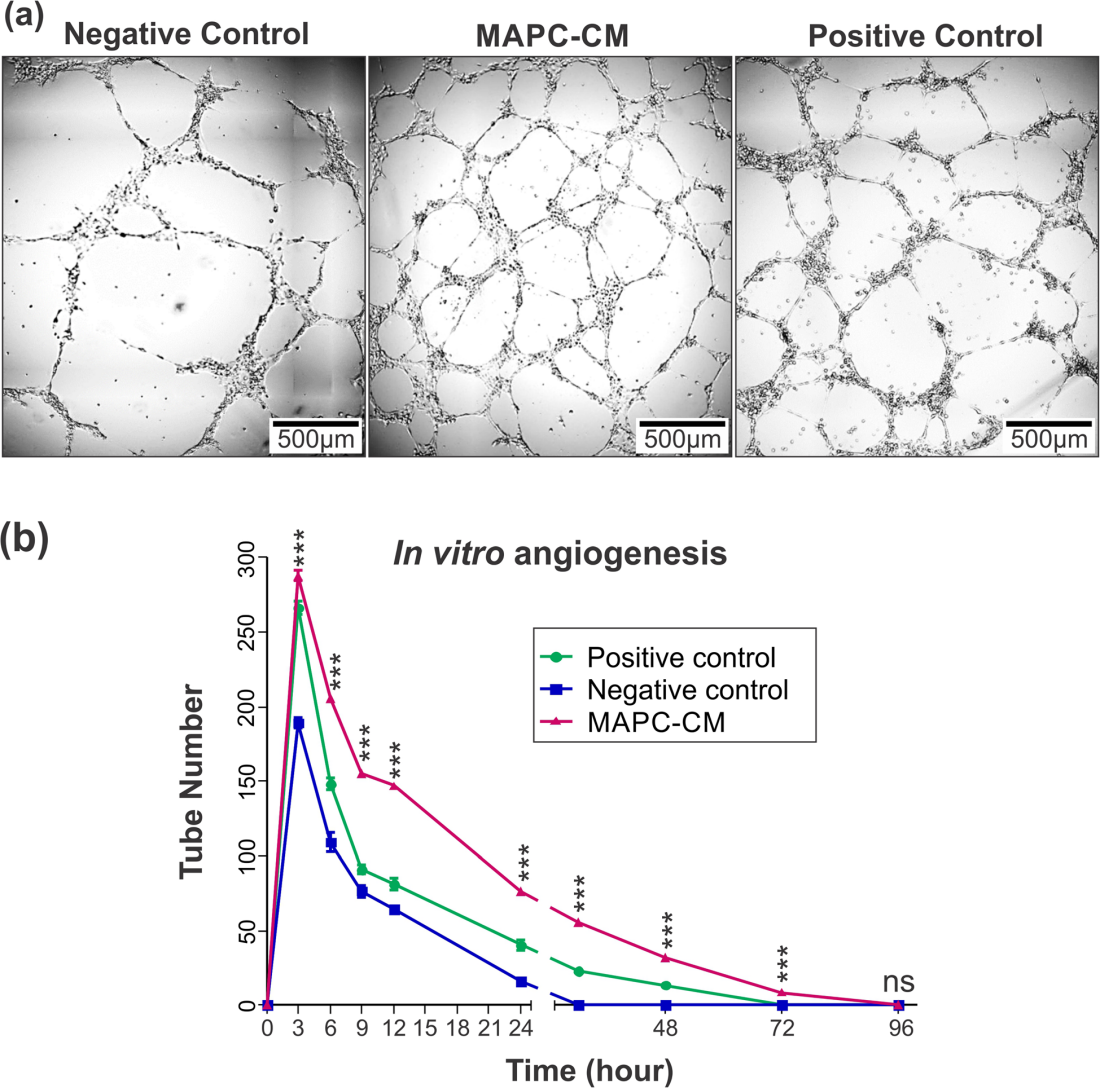
Effect of MAPC-CM on in vitro tube formation. Representative images of HDMEC cells in tube-like structures, 24 h after seeding onto Matrigel (**a**). MAPC-CM encouraged endothelial cells to reorganise into 3D vessel structures at each time point until 72 h (**b**). Values are mean ± SEM of 3 independent experiments. *n* = 6. Statistical significance calculated by one-way ANOVA followed by Tukey test, where * = *p* < 0.05, ** = *p* < 0.01, *** = *p* < 0.001, ns = non-significant

An in vitro tube formation assay was conducted in order to examine the possible effects of MAPC-CM on blood vessel formation. Tube formation of HDMECs was quantified by counting the average number of tube-like structures (Fig. 3a). HDMECs formed the maximum number of blood vessel-like structures in all three groups after 3 h after which tubes began to degrade. As indicated in Fig. 3b, the average number of tube-like structures in each time point was significantly higher in HDMECs incubated with MAPC-CM compared with those incubated with FBS-free DMEM (negative control).

Degradation of tube-like structures began after 3 h, with all structures in FBS-free DMEM (negative control) and DMEM with 10% FBS (positive control) being degraded after 30 and 72 h, respectively. The overall number of tubes remained significantly elevated at each time point in MAPC-CM wells, with a prolonged half-life of tubes observed up to 96 h (Fig. 3b). These results indicate the positive effect of MAPC-CM impact not only on vessel formation but also on vessel stabilisation.

### MAPC-CM enhances collagens I and III deposition of fibroblasts

**Fig. 4:**
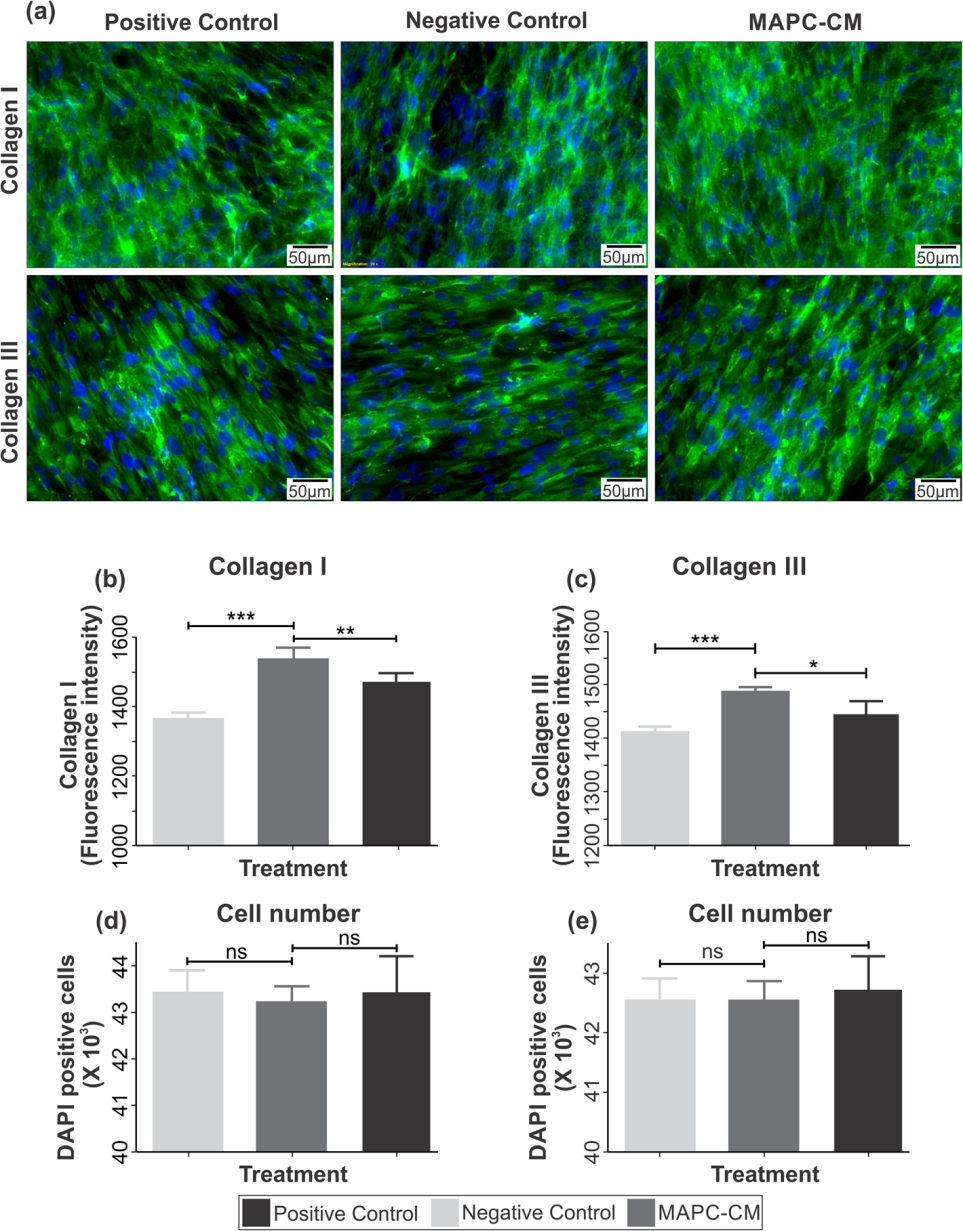
Effect of MAPC-CM on collagens I and III production of HFFs. Representative images of HFFs treated with MAPC-CM versus positive and negative control media stained for collagen I and collagen III (**a**). Fluorescence intensities measured using CellSens dimension software (version 1.12), which show that MAPC-CM increases collagens I and III production by HFFs (**b**, **c**). HFFs were also stained with DAPI and cell numbers in each well were counted (**d**, **e**). Data are presented as mean ± SEM. *n* = 6. Statistical significance calculated by one-way ANOVA followed by Tukey’s test, where * = *p* < 0.05, ** = *p* < 0.01, *** = *p* < 0.001, ns = non-significant

In order to assess the potential effect of MAPC-CM on collagen deposition, cultured HFFs were incubated with MAPC-CM for 60 h prior to staining with collagen I or collagen III antibodies. Cells stained immunocytochemically were imaged on a Zeus microscope (Olympus, Tokyo, Japan) and analysed using cellSense software (Fig. 4a). For intensity measurements, the mean grey intensity value was measured across the well. Collagen I and III expressions quantified as mean grey intensity within the well. The intensity for collagen I was increased by 1.2-fold in cells that were treated with MAPC-CM compared to those that were incubated in FBS-free DMEM (negative control) indicating a significant increase in the collagen I deposition with MAPC-CM treatment (Fig. 4b). The deposition of collagen III by fibroblasts was elevated to 1.05-fold following MAPC-CM stimulation compared to the negative control (Fig. 4c). In both cases, deposition of collagens I and III in response to MAPC-CM treatment were higher than FBS-containing DMEM (positive control) (Fig. 4b, c).

The total number of cells, stained positive with DAPI nuclear stain, were counted to assess the impact of HFF proliferation on the amounts of collagens I and III. The total number of HFFs either in collagen I or collagen III experiment was not significantly different between the cells incubated with MAPC-CM, FBS-free DMEM (negative control) or DMEM with 10% FBS (positive control). This non-significant difference was probably due to the contact inhibition effect, which indicates that the increase in the collagen deposition of HFFs was not due to a corresponding increase in HFF numbers during culture (Fig. 4d, e).

### MAPC-CM treatment improves wound healing in excisional wounds

**Fig. 5:**
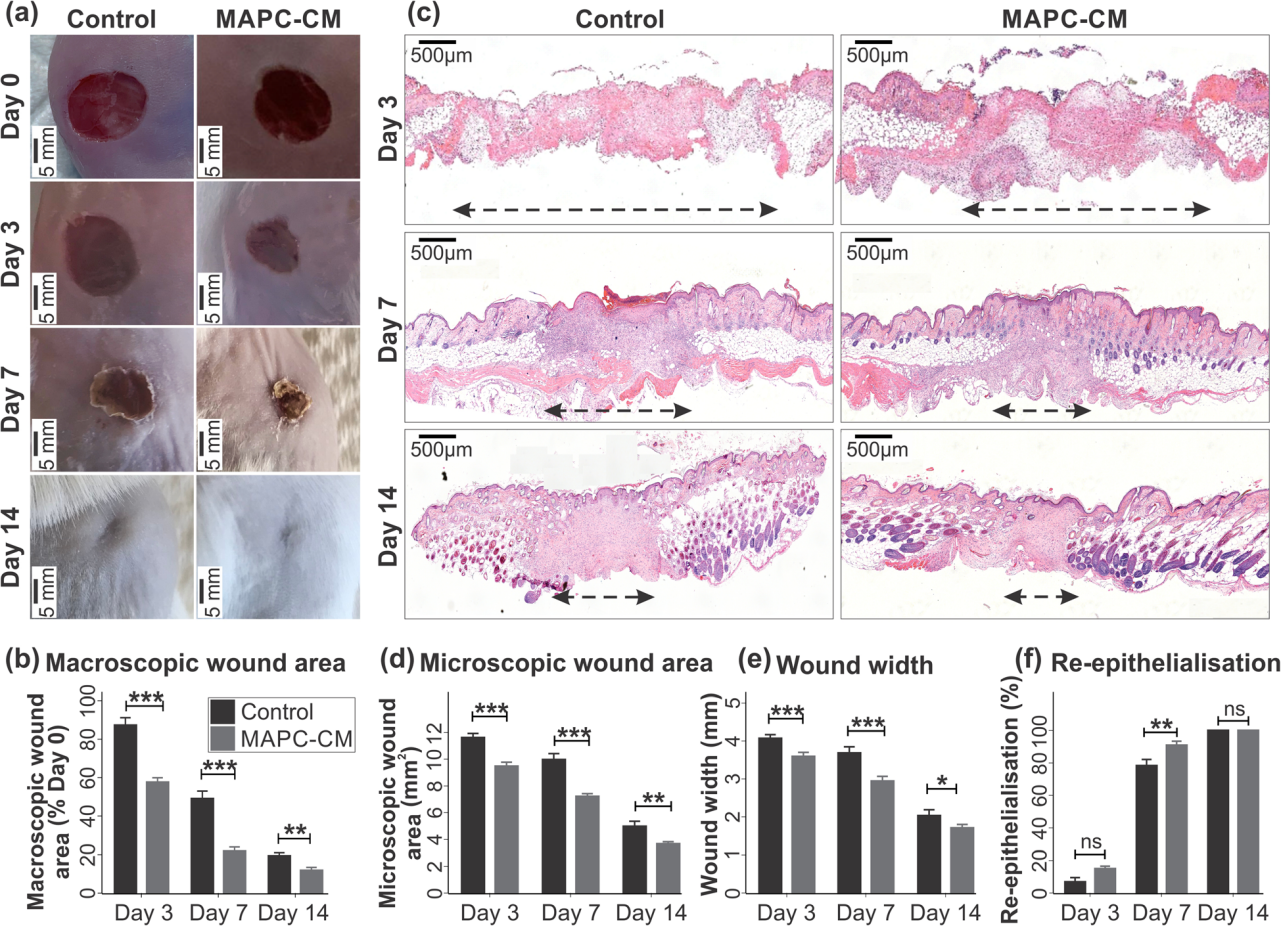
Effect of MAPC-CM on the rate of excisional wound healing. Representative images of MAPC-CM treated and control wounds at days 3, 7 and 14 of healing (**a**). Analysis of images using Image Pro-Plus program demonstrates decreased macroscopic wound area in MAPC-CM-treated wounds on days 3, 7 and 14 post-injury (**b**). Representative images of MAPC-CM-treated and control wounds sections after H&E staining at days 3, 7 and 14 of healing. Images captured at 10× objective. Black dashed lines indicate wound width measured (**c**). The histological analysis of wounds demonstrates decreased microscopic wound area (**d**), wound width (**e**) and increased average re-epithelialisation (**f**) in MAPC-CM-treated wounds. *n* = 8. All data is represented as mean ± SEM. Statistical significance calculated by two-way ANOVA followed by Bonferroni post-tests, where * = *p* < 0.05, ** = *p* < 0.01, *** = *p* < 0.001, ns = non-significant

The effects of MAPC-CM on cutaneous repair and re-epithelialisation was determined using an excisional murine wound healing model. Macroscopic analysis of wounds treated with MAPC-CM showed a significant reduction in average wound area compared to DMEM-treated wounds (control groups) at days 3, 7 and 14 of healing (Fig. 5a, b).

Histological analysis of wound morphology in H&E-stained paraffin-embedded wound tissues (Fig. 5c) also showed decreased microscopic wound area (Fig. 5d) and wound width (Fig. 5e) in MAPC-CM-treated wounds at days 3, 7 and 14 of healing. Further analysis of H&E-stained wound tissues at day 3 of healing revealed 15% re-epithelialisation of the wounds in the MAPC-CM-treated group compared with 6% in the control group. At day 7 of healing, wounds in MAPC-CM-treated mice were 90% re-epithelialised (Fig. 5f). In contrast, wounds from control mice reached only 78% re-epithelialisation by day 7. At day 14 of healing, all wounds in both MAPC-CM-treated and control mice were fully re-epithelialised (Fig. 5f).

### MAPC-CM treatment decreases inflammatory response in excisional wounds

**Fig. 6:**
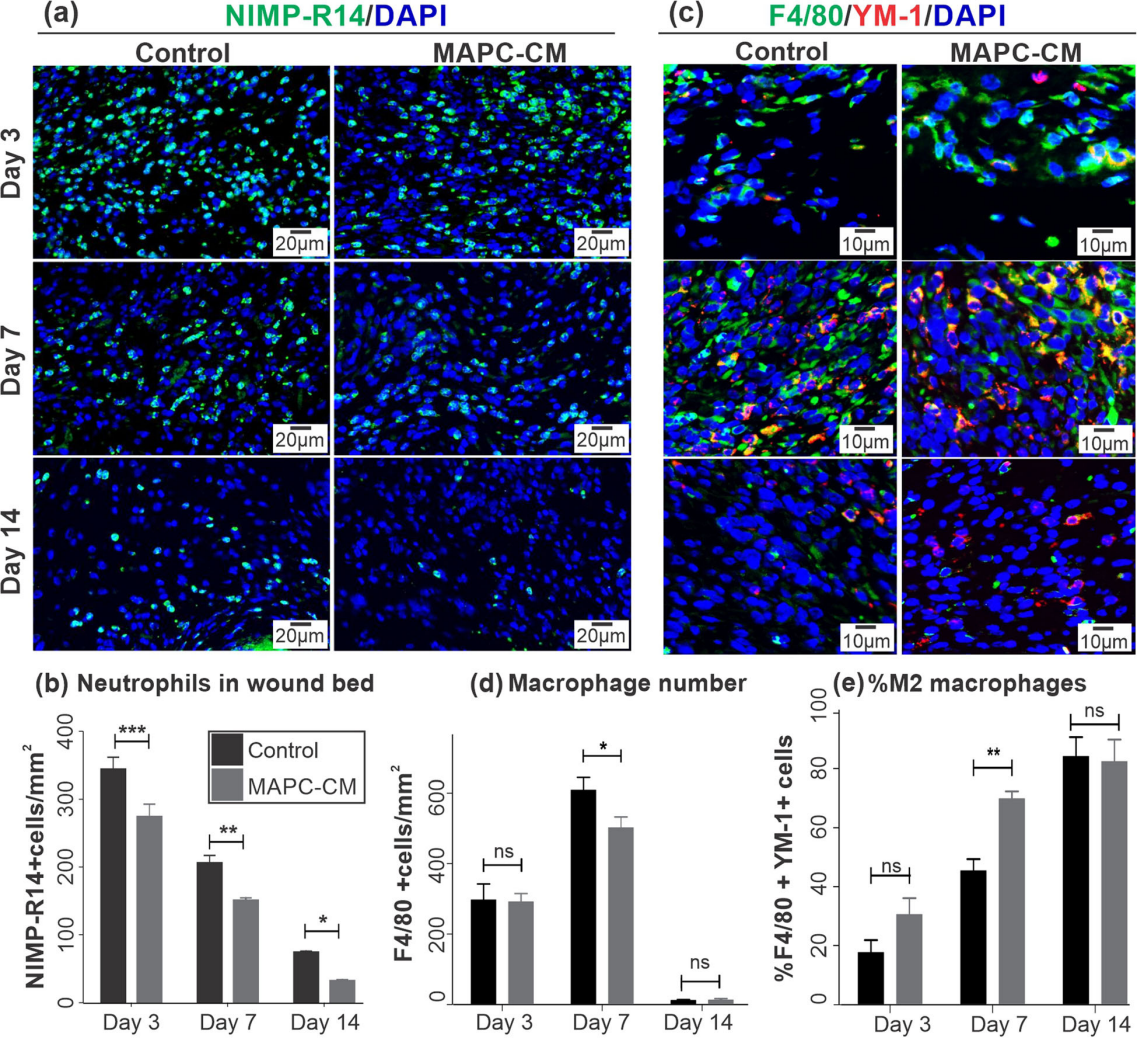
Effect of MAPC-CM on inflammation in excisional wounds. Representative images of immunofluorescent NIMP-R14 positive neutrophils (green) counterstained with DAPI (blue) in wounds at days 3, 7 and 14 post-injury (**a**). Quantification of neutrophil numbers in the wound bed normalised to wound area (**b**). Representative images of immunofluorescent macrophages by F4/80 (green) and YM-1 (red) staining counterstained with DAPI (blue) in wounds (**c**). Quantification of total macrophage number (F4/80+) in the wound bed normalised to wound area (**d**). Quantification of M2 macrophages (F4/80+, YM-1+) as a percentage of total macrophages in the wound bed normalised to the wound area. Images captured at 20× objective. *n* = 8. All data is represented as mean ± SEM. Statistical significance calculated by two-way ANOVA followed by Bonferroni post-tests, where * = *p* < 0.05, ** = *p* < 0.01, *** = *p* < 0.001, ns = non-significant

To explore the impact of MAPC-CM on inflammation, immunofluorescent staining of neutrophil marker NIMP-R14 and macrophage marker F4/80 was carried out to quantify neutrophil and macrophage infiltration into the wound bed. Figure 6a shows fluorescence microscopy images of wounds, where the highest number of neutrophils was observed at day 3 of healing. This is expected as infiltration of neutrophils begins at the early stages of the inflammatory phase [34]. Neutrophil numbers decreased at days 7 and 14 of healing. This reduction can be explained by the fact that immune cells undergo apoptosis to resolve inflammation over the course of healing [34]. Quantification of NIMP-R14-positive cells in the wound area indicated less inflammatory cell infiltration to the wound bed in MAPC-CM-treated mice compared to the control group at days 3, 7 and 14 of healing (Fig. 6b). The quantification of macrophage infiltration using macrophage marker (F4/80) demonstrated a decrease in the presence of F4/80-positive macrophages in MAPC-CM-treated wounds at day 7 (Fig. 6c, d). The percentage of M2 anti-inflammatory macrophages expressing Ym-1 indicated that the rate of polarisation of macrophages to an anti-inflammatory state was increased by MAPC-CM treatment at 7 days of healing (Fig. 6c, e).

### MAPC-CM treatment increases endothelial cell number and angiogenesis in excisional wounds

**Fig. 7:**
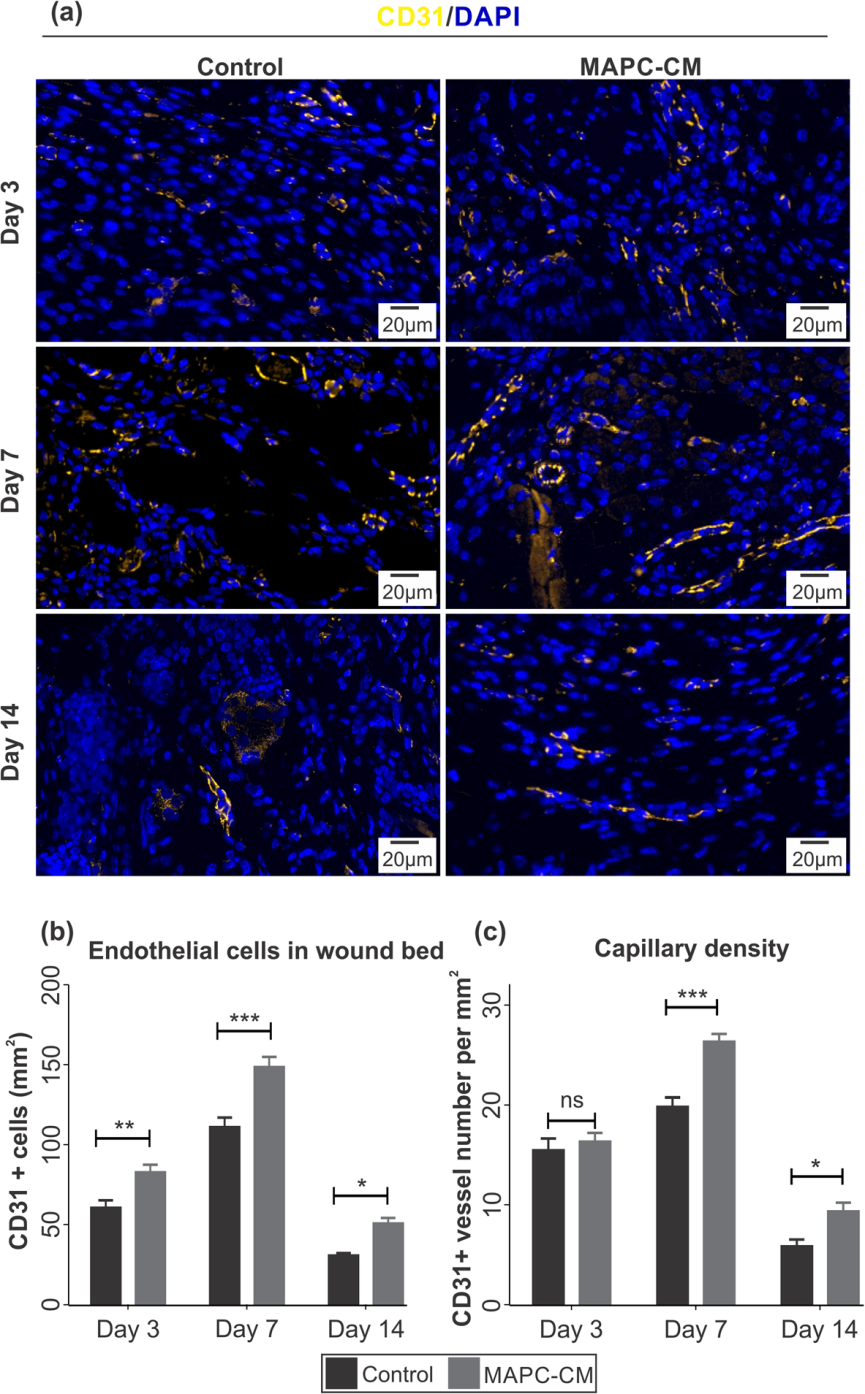
Effect of MAPC-CM on angiogenesis in excisional wounds. Representative images of immunofluorescent detection of endothelial cells and blood vessels by CD31 staining (yellow) counterstained with DAPI (blue) at wounds. Images captured at 20× objective (**a**). Quantification of endothelial cells in the wound bed normalised to the area (**b**). Quantification of blood vessels in the wound bed normalised to the area (**c**). *n* = 8. All data is represented as mean ± SEM. Statistical significance calculated by two-way ANOVA followed by Bonferroni post-tests, where * = *p* < 0.05, ** = *p* < 0.01, *** = *p* < 0.001, ns = non-significant

In order to investigate the effect of MAPC-CM on angiogenesis, the tissues were immunostained with endothelial cell marker, CD31 (Fig. 7a). The number of CD31-positive endothelial cells and blood vessels was increased from day 3 to day 7 but reduced at day 14. Increased numbers of CD31-positive endothelial cells in MAPC-CM-treated wounds were observed at each time point when compared to the control group (Fig. 7b). Capillary density, as measured by the number of mature vessels per mm^2^, was also significantly increased in MAPC-CM-treated wounds at days 7 and 14 of healing (Fig. 7c).

### MAPC-CM treatment elevates collagen expression in excisional wounds

**Fig. 8:**
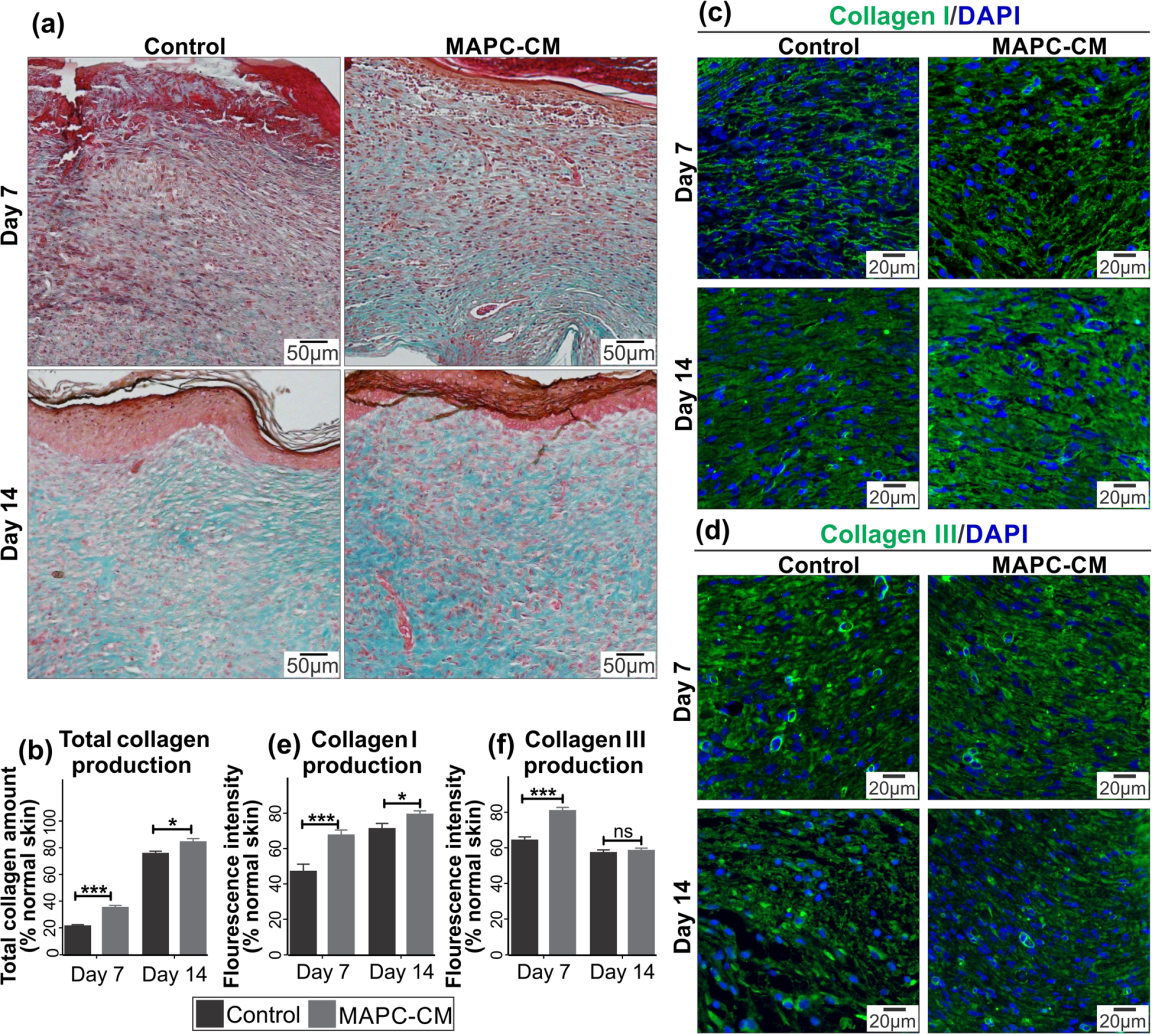
Effect of MAPC-CM on total collagen production and collagens I and III expression in excisional wounds. Representative images of collagen detection (green) by Masson’s trichrome staining Images captured at 10× objective (**a**). Quantification of total collagen levels in wound bed normalised to the normal skin collagen (**b**). Representative images of immunofluorescent detection of collagen I and collagen III (green) counterstained with DAPI. Images captured at 20× objective (**c**). Quantification of collagen I expression as mean grey intensity within the wound bed and normalised to the normal skin collagen I level (**d**). Quantification of collagen III expression as mean grey intensity within the wound bed and normalised to the normal skin collagen III level (**e**). Values are mean ± SEM of 3 independent experiments. *n* = 6. Statistical significance calculated by two-way ANOVA followed by Bonferroni post-tests, where * = *p* < 0.05, ** = *p* < 0.01, *** = *p* < 0.001, ns = non-significant

Masson’s trichrome staining was undertaken to measure the total amount of collagen in wound tissues (Fig. 8a). The analysis of stained sections revealed that total collagen in MAPC-CM-treated wounds on day 7 and day 14 was elevated significantly compared to control wounds (Fig. 8b).

In order to investigate the effect of MAPC-CM on the expression of different collagen types, the wound tissues were stained with collagen I and collagen III antibodies (Fig. 8c). Results show that both collagens I and III expression was increased at day 7 in MAPC-CM-treated wounds. Collagen I expression was also elevated in MAPC-CM-treated wounds at day 14 of healing while there was no significant difference in collagen III expression between two groups at day 14 of healing (Fig. 8d, e).

## Discussion

Over the last two decades, stem cells have been extensively used in regenerative medicine due to their positive effects on cardiac, bone and liver regeneration [7]. However, the clinical use of these cells is limited due to the barriers that exist in utilising stem cells including low differentiation capability, cellular senescence and limited replicative lifespan. The translation of stem cell therapies into clinical products has further been limited due to low cell survival and poor engraftment, immunogenicity and tumorigenicity of cells after administration [35] Recent studies have suggested that stem cells secrete biomolecules into their environments that are beneficial for inducing cell differentiation and functional responses important for tissue repair [11].

MSCs have been previously shown to have paracrine effects on regulating angiogenesis, promoting tissue remodelling and recruiting other cells as well as having immunomodulatory, anti-inflammatory and anti-fibrosis effects [36–39]. Although MAPC cells and MSCs have some similarities, in that both are bone marrow-derived adherent and non-haematopoietic stem cells [14], their secretome is different as the composition and therapeutic benefit of the secretome can be impacted by isolation methods, culture and storage conditions [40].

In the present study, treatment of excisional wounds with MAPC-secretome in the form of MAPC-CM led to improved wound healing outcomes evidenced by decreased wound area, wound width and inflammatory cell infiltration as well as increases re-epithelialisation. Furthermore, MAPC-CM-treated wounds displayed reduced inflammation, increased angiogenesis and increased amounts of collagens I and III.

MAPC cells possess broader expansion capacities than MSCs and do not show signs of cell senescence, karyotypic abnormalities and telomere shortening [41]; these facilitate large scale production of MAPC cells and MAPC-secretome for therapeutic treatment.

Previous studies have reported the effectiveness of live MAPC cell treatment on wound healing in mice [42–44]. Herein, MAPC-CM is shown to be equally as effective as MAPC cell treatment with macroscopic and histological analyses of wound tissues confirming enhanced wound closure time and increased rate of re-epithelialisation. This is consistent with the in vitro studies showing a clear beneficial effect of MAPC-CM on keratinocyte and fibroblast proliferation and migration. Analysis of MAPC-CM suggests that its effect could be partly due to the presence of secreted proteins that have a significant impact on the proliferation and migration of keratinocytes and fibroblasts including FGF-2, MMP-1 and HGF [23].

MAPC cells have previously been reported to have immunomodulatory and anti-inflammatory potential both in in vitro culture and in recipients [45]. In the present study, the analysis of NIMP-R14-stained wound tissues suggested there was reduced inflammation in the MAPC-CM-treated wounds evidenced by a significant reduction in neutrophilic infiltration. In addition, the number of macrophages in wounds were also lower in MAPC-CM-treated wounds. The polarisation of inflammatory M1 macrophages to anti-inflammatory M2 macrophages was also promoted in MAPC-CM-treated wounds. The anti-inflammatory effect may be explained by the secretion of cytokines and growth factors by MAPC cells into the conditioned media that contribute to inflammatory cell infiltration and resolution. Analysis of the bead array assays confirmed that MAPC-CM contains high concentrations of inflammatory cytokines mainly IL-6 (both pro and anti-inflammatory cytokine). Our findings are also consistent with other studies that have confirmed the secretion of various pro/anti-inflammatory cytokines including IL-6, IL-10, IL1α, IL8 and TNFα by MAPC cells [19].

In the proliferation phase of wound healing, fibroblasts migrate to the wound site and secrete components of ECM mainly collagens I and III within the wound bed. During the remodelling phase, the relative proportions of collagens I and III changes (type I collagen content increases while collagen type III decreases), and total collagen content increases. These functions are all required for wound contraction [2, 46]. MAPC-CM was found to contain secreted collagens, ECM proteins and other growth factors which mediate ECM production and remodelling [19]. In this study, we also have found that the production of collagens I and III by dermal fibroblasts was augmented when they were treated with MAPC-CM in vitro. Additionally, measuring collagen expression in mice treated with MAPC-CM showed an increase in the production of both collagen I and III at day 7 and collagen I production at 14 post-injury while there was no difference in the amount of collagen III at day 14. This result confirmed the improvement of collagen remodelling in MAPC-CM-treated wounds. This improvement could be partly due to the presence of FGF-2 in MAPC-CM, which has been shown to induce collagen production [47].

Failure of neovascularization and angiogenesis is another important factor which contributes to impaired wound healing [48]. Our results revealed an increase in proliferation and migration in endothelial cells in the presence of MAPC-CM. Endothelial cells treated with MAPC-CM also formed more vessel-like tubes than untreated ones. Furthermore, formed tubes persisted in culture for longer when treated with MAPC-CM. Accordingly, the number of endothelial cells and blood vessels was also increased in MAPC-CM-treated wounds which shows the significant effects of MAPC-CM on angiogenesis and wound healing. The number of endothelial cells and blood vessels were increased in MAPC-CM-treated wounds which shows the significant effects of MAPC-CM on angiogenesis and wound healing. We also showed that MAPC-CM contains VEGF which is a proangiogenic factor with an ability to stimulate proliferation, migration and vessel formation of endothelial cells [19, 49].

Overall, histological and immunohistochemistry observations, taken together with the morphometric parameters, demonstrated that wounds treated with MAPC-CM show improved healing responses. This improvement was mediated through a combination of reduced inflammation and enhanced re-epithelialisation, angiogenesis and collagen production. This study suggests the use of MAPC-CM as a promising alternative to stem cell-based therapies for the treatment of wounds.

## Conclusion

Intradermal administration of MAPC cell secretome appears to be safe and enhances cutaneous wound healing by promoting skin cell proliferation and migration, balancing inflammation and improving the formation of extracellular matrix and angiogenesis. These results provide preclinical evidence that supports the translation of stem cell-free therapy for the treatment of wounds.

## Supplementary information

**Additional file 1.**

## Data Availability

The data that support the findings of this study are available from the corresponding author upon reasonable request.

## Abbreviations

ALCAM: Activated leukocyte cell adhesion molecule
CD31: Cluster of differentiation 31
DAPI: 4′,6-Diamidino-2-phenylindole
DMEM: Dulbecco’s modified Eagle’s medium
ECM: Extracellular matrix
FBS: Foetal bovine serum
FGF-2: Fibroblast growth factor-2
H&E: Haematoxylin and eosin
HDMECs: Human dermal microvascular endothelial cells
HFFs: Human foreskin fibroblasts
HGF: Hepatocyte growth factor
IL: Interleukin
MAPC cells: Multipotent adult progenitor cells
MAPC-CM: Multipotent adult progenitor cells-conditioned medium
MCP-1: Monocyte Chemoattractant Protein-1
MMP-1: Matrix metalloproteinase
MSCs: Mesenchymal stem cells
NGS: Normal goat serum
PBS: Phosphate-buffered saline
TIMP-1: Tissue inhibitor of metalloproteinase
TNF-α: Tumour necrosis factor alpha
VCAM: Vascular cell adhesion protein
VEGF: Vascular endothelial growth factor
