# Supplementary material for: Human multipotent adult progenitor cell-conditioned medium improves wound healing through modulating inflammation and angiogenesis in mice
Source: Stem Cell Res Ther. 2020 Jul 17;11:299. doi: 10.1186/s13287-020-01819-z (PMC7368692; doi:10.1186/s13287-020-01819-z)
Supplement: Supplementary file 1 — Additional file 1. [file 13287_2020_1819_MOESM1_ESM.zip › Supplementary Information.docx]

**Supplementary Information**

**qRT-PCR**

The specific gene expression of MAPC cells and donor matched MSCs was determined using qRT-PCR. Total RNA was extracted using AllPrep RNA Isolation Kit based on the manufacturer’s protocol (QIAGEN GmBH, Hilden, Germany). First-strand cDNA was synthesized in a 20 μL reaction from 1 μg total RNA by Bio-Rad iScript kit (BioRad, NSW, Australia). The conditions for the cDNA synthesis were: 5 minutes at 25°C, 30 minutes at 42°C, 5 minutes at 80°C, and cooling down to 4°C. The qRT-PCR was carried out using SsoAdvanced™ Universal SYBR® Green Supermix, synthesis (BioRad, NSW, Australia). The expression of 5 genes were quantified and compared between MAPC cells and donor matched MSCs (Table 1). For the qRT-PCR run, each well contained 7.5 µL NFH_2_O, 200 nm primer pairs (of each forward and reverse primers), 10 µL of SsoAdvanced™ Universal SYBR® Green Supermix, and 1 µL of synthesized cDNA template. The plate was run through the amplification cycle at the PCR cycler which began with 30 seconds at 90°C, followed by 32 repeating steps of 95°C for 5 seconds and 60°C for 20 seconds with the SYBR green signal read after each annealing step. The tubes were then cooled to 4°C. A melt curve was obtained while increasing from 65 to 95°C in 0.5 increments every 5 seconds to check the specificity of amplified fragments.

**Table 2: Primer sets for characterising MAPC using qRT-PCR**

| **Markers** | **Forward primer** | **Reverse primer** | **Expected Expression** |
| --- | --- | --- | --- |
| ANGPTL2 | 5'-CCTGGATGGCTCTGTTAACTTC-3' | 5'-GTTTGTAGTTGCCTTGGTTCG-3' | Higher expression in  MSCs |
| PTGS1 | 5'-TTCGGTGTCCAGTTCCAATAC-3' | 5'-TGACATCCACAGCCACATG-3' | Higher expression in  MAPC |
| CXCL5 | 5'-TCTGCAAGTGTTCGCCATAG-5' | 5'-CAGTTTTCCTTGTTTCCACCG-3' | Expression in MAPC/  No expression in MSCs |
| INSC | 5'-GTTGAATGCCATCCGTGTTC-3' | 5'-GTCAGAGGCACACTGTTCTAG-3' | Higher expression in  MAPC |
| AGT | 5'-CTGATCCAGCCTCACTATGC-3' | 5'-AGGTCATAAGATCCTTGCAGC-3' | Expression in MSC/  No expression in MAPC |
| ATP5B | 5'-GATCCTCTAGACTCCACCTCTC 3' | 5'-AGAAAGTTCATCCATACCCAGG-3' | Metabolic Marker,  Equal expression |

**MAPC characterisation**

A panel of PCR markers was used to compare the human MAPC with donor matched MSCs in term of transcriptional profile and also to confirm that the MAPC used in this study maintained their phenotype at the point of use. The qRT-PCR confirmed that there was higher expression of ANGPTL2 gene in MSCs while the expression of PTGS1 and INSC were higher in MAPC. CXCL5 was only expressed in MAPC whereas AGT was only detected in MSCs (Fig. S1). This transcriptome analysis demonstrated that MAPC and their donor matched MSCs are two distinct cell populations, with each expressing different characteristic genes that are associated with the functional ability of the cells.

**Supplementary Figure Legend**

**Fig S1:** Phenotype differences between MAPC cells and MSCs. qRT-PCR analysis was used to distinguish MAPC and MSCs derived from same donor based on the differences in characteristic genes expression. n=6. Results are representative of three independent experiments.
