# Supplementary figures and images for: Human multipotent adult progenitor cell-conditioned medium improves wound healing through modulating inflammation and angiogenesis in mice
Source: Stem Cell Res Ther. 2020 Jul 17;11:299. doi: 10.1186/s13287-020-01819-z (PMC7368692; doi:10.1186/s13287-020-01819-z)

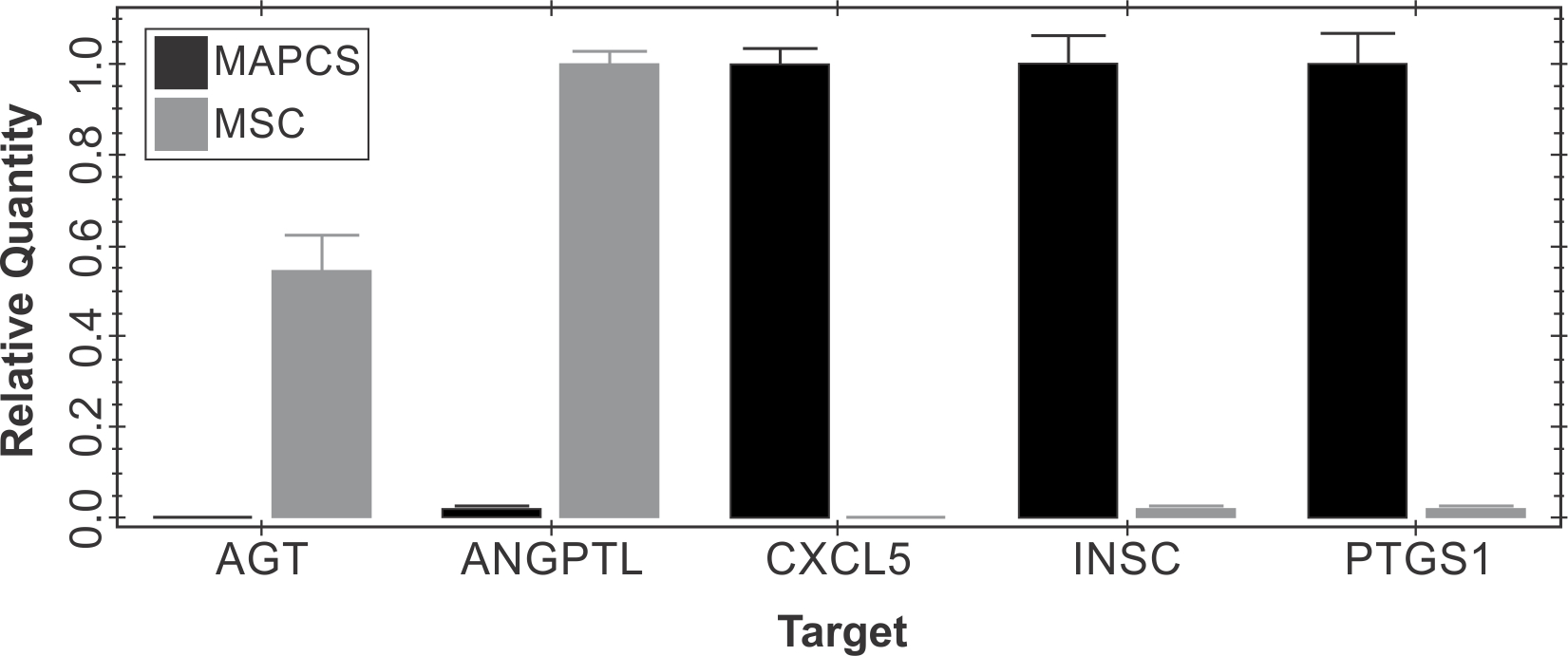

Supplement: Supplementary file 1 — Additional file 1. [file 13287_2020_1819_MOESM1_ESM.zip › Fig S1.jpg]
